# Effect of high-flow nasal cannula at different flow rates on diaphragmatic function in subjects recovering from an acute exacerbation of COPD: a physiological prospective pilot study

**DOI:** 10.1186/s44158-024-00173-3

**Published:** 2024-06-24

**Authors:** Nicolás Colaianni-Alfonso, Iván Castro, Vanesa Cáceres, Guillermo Montiel, Salvatore Maurizio Maggiore, Luigi Vetrugno

**Affiliations:** 1 Respiratory Intermediate Care Unit, Hospital Juan A. Fernández, Ciudad Autónoma de Buenos Aires, Buenos Aires, C1425 Argentina; 2 Department of Anesthesiology, Critical Care Medicine and Emergency, Department of Innovative Technologies in Medicine and Dentistry, “G. d’Annunzio” University Chieti-Pescara, Chieti, Italy; 3 Department of Medical, Oral and Biotechnological Sciences, University of G. d’ Annunzio, Chieti-Pescara, Italy

**Keywords:** Chronic obstructive pulmonary disease, Diaphragm ultrasound, High-flow nasal cannula, Noninvasive ventilation, Respiratory failure

## Abstract

**Background:**

Noninvasive ventilation (NIV) is widely employed as the initial treatment for patients with chronic acute exacerbation of obstructive pulmonary disease (AECOPD). Nevertheless, high-flow nasal cannula (HFNC) has been increasingly utilized and investigated to mitigate the issues associated with NIV. Flow rate may play a significant role in diaphragmatic function among subjects recovering from AECOPD. Based on these observations, we conducted a physiological study to assess the impact of HFNC therapy on diaphragmatic function, as measured by US, respiratory rate (RR), gas exchange, and patient comfort at various flow rates.

**Methods:**

A prospective physiological pilot study enrolled subjects with a diagnosis of AECOPD who required NIV for more than 24 h. After stabilization, these subjects underwent a 30-min trial using NIV and HFNC at different sequential flow rates (30–60 L/min). At the end of each trial, diaphragmatic displacement (DD, cm) and diaphragmatic thickness fraction (DTF, %) were measured using ultrasound. Additionally, other physiological variables, such as RR, gas exchange, and patient comfort, were recorded.

**Results:**

A total of 20 patients were included in the study. DD was no different among trials (*p* = 0.753). DTF (%) was significantly lower with HFNC-30 L/min compared to HFNC-50 and 60 L/min (*p* < 0.001 for all comparisons). No significant differences were found in arterial pH and P_a_CO_2_ at discontinuation of NIV and at the end of HFNC trials (*p* > 0.050). During HFNC trials, RR remained unchanged without statistically significant differences (*p* = 0.611). However, we observed that HFNC improved comfort compared to NIV (*p* < 0.001 for all comparisons). Interestingly, HFNC at 30 and 40 L/min showed greater comfort during trials.

**Conclusions:**

In subjects recovering from AECOPD and receiving HFNC, flows above 40 L/min may not offer additional benefits in terms of comfort and decreased respiratory effort. HFNC could be a suitable alternative to COT during breaks off NIV.

## Introduction

Noninvasive ventilation (NIV) is widely employed as the initial treatment for patients with chronic acute exacerbation of obstructive pulmonary disease (AECOPD) who experience acute hypercapnic respiratory failure caused by different triggers [1]. In these subjects, the application of NIV has demonstrated efficacy in improving respiratory gas exchange, reducing the need for endotracheal intubation (ETI), and enhancing overall survival [2, 3]. Nevertheless, high-flow nasal cannula (HFNC) has been increasingly utilized and investigated to mitigate the issues associated with NIV, including interface discomfort, pressure injury, sleep disturbances, and patient-ventilator asynchrony [4, 5]. HFNC facilitates secretion clearance, prevents epithelial injury, and mitigates airway inflammation [6]. HFNC delivers positive airway pressure, countering intrinsic positive end-expiratory pressure (PEEPi) and thereby reducing the isometric workload linked with dynamic hyperinflation. However, some patients may experience heightened inspiratory effort as flow rates escalate, potentially stemming from discomfort, increased dynamic hyperinflation, or elevated expiratory resistance [7].

Recent meta-analyses in COPD found that HFNC significantly reduces P_a_CO_2_ compared to conventional oxygen therapy (COT) [8–10]. On the other hand, conflicting results are observed in patients with AECOPD, one meta-analysis concluded no significant benefit in reducing ETI compared with NIV [11], while another indicated that HFNC is non-inferior to NIV in decreasing the risk of ETI during AECOPD [12]. HFNC has some physiological advantages for AECOPD patients: heated and humidified gas delivery, anatomical dead space washout, “PEEP” (positive end-expiratory pressure) effect, provision of stable inspired oxygen fraction (F_i_O_2_), and treatment comfort [13]. In addition, HFNC allows us to combine with vibrating mesh nebulizers to deliver aerosol therapy without impairing the performance of respiratory support [14].

Although the physiological effects of HFNC are well known, few studies have assessed the diaphragmatic function in subjects recovering from an AECOPD treated with HFNC to individualize this treatment [15, 16]. Ultrasonography (US) is a simple tool available at the bedside that allows the evaluation of diaphragm function, present or abolished, through M-mode, with the measurement of diaphragmatic displacement (DD, cm) and its force through the measurement of diaphragm thickening fraction (DTF, %) [17–19]. Both measures are affected in various ways by subjects admitted to the emergency department with AECOPD [20, 21]. Flow rate may play a significant role in diaphragmatic function among subjects recovering from an AECOPD. Based on these observations, we conducted a physiological study to assess the impact of HFNC therapy on diaphragmatic function, as measured by US, respiratory rate (RR), gas exchange, and patient comfort at various flow rates.

## Methods

### Study design

This prospective physiological pilot study was carried out at the respiratory intermediate care unit (RICU) of the Hospital General de Agudos Juan A. Fernández, Buenos Aires, Argentina, from March 2022 to March 2023. The institutional review boards reviewed the protocol and authorized prospective data collection (identified code no. 2663). Written informed consent to participate was obtained from each subject or their relatives.

### Subjects

Patients with a previous diagnosis of COPD who were admitted to the RICU with AECOPD and required NIV for acute hypercapnic respiratory failure (pH ≤ 7.35 with a P_a_CO_2_ ≥ 45 mmHg) [22]. Underlying COPD could be documented by spirometry and defined by an FEV_1_/FVC < 0.70 or, alternatively, highly suspected underlying COPD. Subjects with suspected underlying COPD without previous spirometry should have a history of smoking and emphysema on chest radiograph or computed tomography scan without other reasons for respiratory acidosis [23].

After initial management and stabilization with NIV, patients were eligible for inclusion in the study. Inclusion criteria were as follows: (1) NIV duration exceeding 24 h, (2) full patient cooperation, (3) arterial pH ≥ 7.35 during NIV, (4) RR ≤ 30 breaths per minute, and (5) clinical stability, indicated by the absence of dyspnea measured on a visual analog scale (VAS), the absence of pain, agitation, and fever.

Exclusion criteria were as follows: (1) Diaphragm paralysis, (2) clinical signs of distress or respiratory muscle fatigue, (3) hemodynamic instability (systolic arterial pressure < 90 mmHg or mean arterial pressure < 60 mmHg or requirement of vasoactive agents), (4) cardiac arrhythmia, (5) impaired renal function, and (6) NIV intolerance.

### Study protocol

**Fig. 1:**
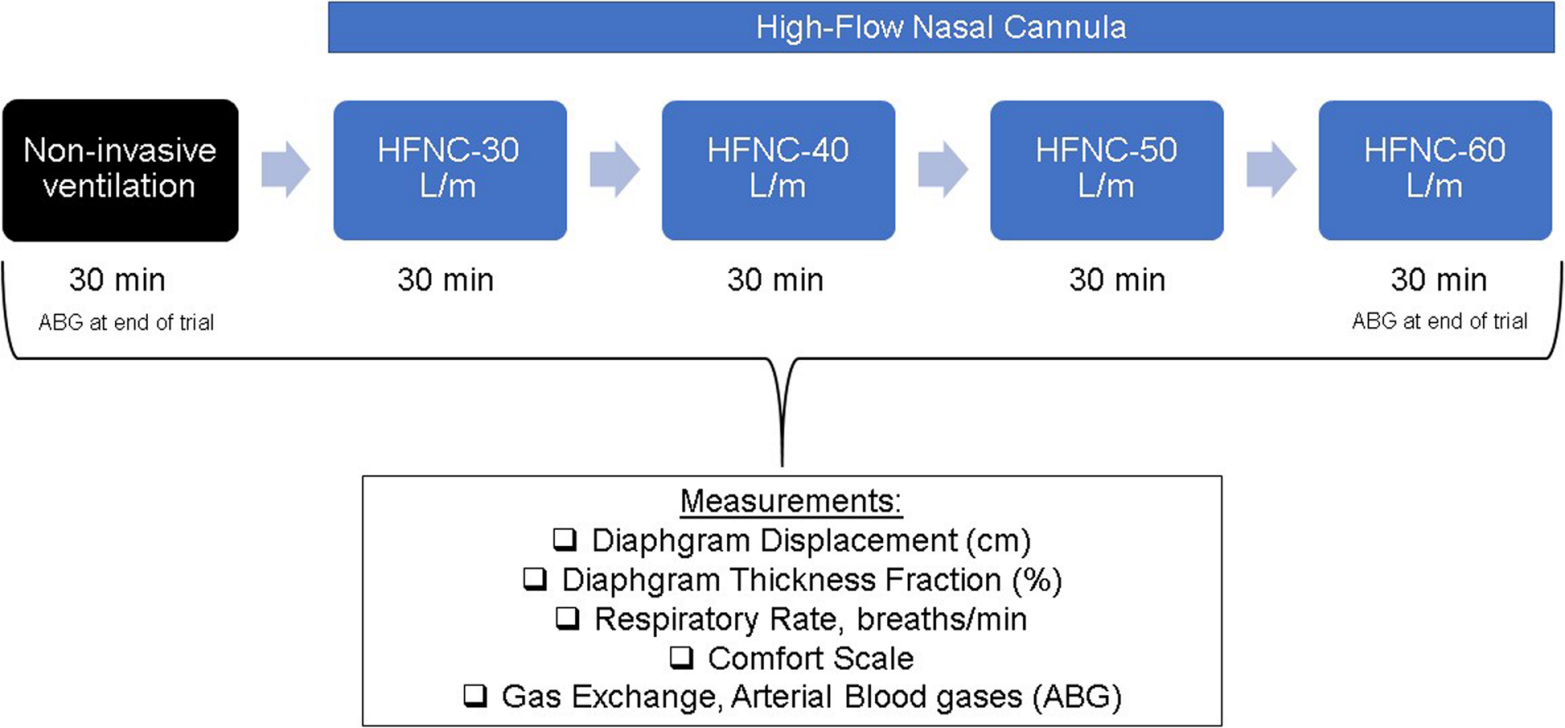
Study protocol

After enrollment, all patients underwent five 30-min trials sequentially, as outlined in the study protocol illustrated in Fig. 1. The first trial utilized NIV delivered by a dedicated ventilator (Astral 150, ResMed, San Diego, CA, USA) equipped with a low-pressure oxygen source via a non-vented face mask with a blue elbow and double-limb circuit (FreeMotion RT041, Fisher and Paykel, Auckland, New Zealand). Subsequently, patients were transitioned to HFNC using standard devices (Airvo 2, Fisher and Paykel, Auckland, New Zealand) with a medium-sized cannula. Flow rates of 30, 40, 50, and 60 L/min were sequentially administered, with a temperature set to 34 °C. F_i_O_2_ was adjusted to maintain oxygen saturation measured by pulse oximetry (S_p_O_2_) 88–92% and kept constant throughout the protocol. We also encouraged patients to breathe with their mouths closed as often as possible to enhance the maximum effect of HFNC.

Patients remained on HFNC following completion of the study. However, if agitation or alterations in mental status, use of respiratory accessory muscles, paradoxical motion of the abdomen, dyspnea, pH < 7.30, or hemodynamic instability (systolic blood pressure < 90 mmHg or > 180 mmHg) occurred, the protocol was discontinued.

### Data collection

Upon admission, we documented demographic data including age, gender, body mass index (BMI), long-term oxygen therapy, Global Initiative for Chronic Obstructive Lung Disease (GOLD) classification, comorbidities, Acute Physiology and Chronic Health Evaluation II (APACHE II) score, Glasgow Coma Scale, vital signs, and arterial blood gases (ABG) at admission. We also recorded NIV settings and the number of days until clinical stability.

### Diaphragmatic ultrasonography

At the end of each trial, after excluding the presence of a hemidiaphragm chronic elevation or paralysis with ultrasound, two investigators (I. C. and V. C.), unaware of the study aims, independently performed bedside sonographic evaluation of the right hemidiaphragm. The following ultrasound device was used: a Philips Lumify® ultrasound machine (Philips Medical Systems, Bothell, WA, USA) with a convex transducer, a Sonoscape S6® ultrasound machine (Yizhe Building, Yuquan Road, Shenzhen, 518,051, China).

Diaphragm displacement (DD [cm]) was assessed on the right side by using the subcostal view in B-mode and transverse scanning. We use right-side approach as the restricted acoustic window presented by the spleen and gastric bubble on the left side. This limitation is attributed to the swift inspiratory lung movement observed in dyspneic subjects and the acoustic impediment posed by air within the bowel and stomach [19, 24]. The US measurements were always performed during spontaneous breathing. DTF was assessed at both end-expiration (DTF-exp [mm]) and end-inspiration (DTF-insp [mm]) phases. The DTF, serving as an indirect measure of diaphragmatic effort [24–26], was computed using the following formula: DTF (%) = ([DTF-insp — DTF-exp]/DTF-exp) × 100. Compared with DD, DTF (%) is a more sensitive and qualitatively accurate parameter and provides a more comprehensive measure of diaphragm contraction. For each patient, three evaluations of DD were conducted on the right side, and three measurements of expiratory and inspiratory DTF were taken on the right side. The average values for DD, as well as expiratory and inspiratory thickness, were calculated. Two operators performed all measurements for each participant.

### Other physiological variables

We evaluated gas exchange by ABG at NIV discontinuation and at the end of HFNC trials. RR was measured during the trials, and patient comfort was assessed using the visual analog scale (VAS) (0 = representing the worst possible comfort and 10 = representing no discomfort).

### Simple sizes calculation

Given the physiological design of the study, we did not conduct a formal sample size calculation. Consistent with previous investigations employing similar designs on the topic [7, 27], we aimed to enroll 20 subjects. This sample size was deemed adequate for drawing meaningful conclusions on these endpoints.

### Statical analysis

The normality of data distribution was assessed by the Shapiro–Wilk test. Normally distributed variables are expressed as mean ± standard deviation and were analyzed by repeated measures analysis of variance (ANOVA) followed by a post hoc pairwise comparison with Bonferroni adjustment. Non-normally distributed variables are expressed as median and interquartile range (IQR) and were compared by Friedman’s two-way ANOVA by ranks with a Dunn’s test post hoc pairwise comparison with Bonferroni correction. Categorical variables are expressed as frequency and percentage. The reproducibility of US measurements was expressed as the intra-class correlation coefficient (ICC). The coefficient of repeatability was calculated as the British Standards Institution repeatability coefficient (twice the standard deviation of the differences in repeated measurements). A *p*-value of less than 0.05 was considered statistically significant. The statistical analysis was performed using R Studio (Version 1.3.1093, R Foundation for Statistical Computing, Vienna, Austria).

## Results

### Patients’ characteristics

A total of 20 patients were included in the study, with a median age of 65 (interquartile range [IQR], 62–71) years. Fifteen (75%) of these were men. Regarding clinical characteristics, the subjects had a median body mass index (BMI) of 27.6 (19.4–31.9 kg/m^2^) and a median APACHE II score of 15 (9–17) points. Fifteen subjects (60%) were classified as GOLD IV. The patients’ characteristics are listed in Table 1.

**Table 1:** Demographic and baseline characteristics of subjects with COPD exacerbation admitted to respiratory intermediate care unit

Variables	*n* = 20
Age, years	65 (62–71)
Male gender, *n* (%)	15 (75)
BMI, kg/m^2^	27.6 (19.4–31.9)
LTOT, *n* (%)	5 (25%)
GOLD classification, *n* (%)	
III	5 (25)
IV	15 (60)
Vital signs at admission	
Respiratory rate, breaths/min	30 (24–35)
Heart rate, beats/min	90 (86–97)
S_p_O_2_, %	85 (84–87)
Comorbidities, *n* (%)	
Hypertension	14 (70)
Cardiovascular disease	5 (25)
Chronic kidney disease	4 (20)
Diabetes	3 (17)
APACHE II, score	15 (9–17)
Glasgow Coma Scale, points	15 (15–15)
ABG at admission	
Arterial pH	7.32 (7.26–7.34)
P_a_CO_2_, mmHg	62 (55–65)
P_a_O_2_, mmHg	59 (55–65)
HCO3, mmol/L	30.05 (28.70–32.17)
NIV setting at admission	
PSV, cmH2O	10 (8–12)
PEEP, cmH2O	7 (6–8)
Tidal volume, mL	440 (340–489)
F_i_O_2_, %	35 (30–42)
Vital signs at clinical stability	
Respiratory rate, breaths/min	21 (20–23)
Heart rate, beats/min	83 (75–92)
SpO2, %	92 (88–93)
ABG at NIV discontinuation	
Arterial pH	7.39 (7.37–7.42)
P_a_CO_2_, mmHg	55 (48–66)
P_a_O_2_, mmHg	65 (63–72)
HCO3, mmol/L	29.30 (27.40–31.15)
Days until clinical stability	2 (1–3)
ABG at the end of HFNC trials	
Arterial pH	7.40 (7.38–7.42)
P_a_CO_2_, mmHg	53 (48–63)
P_a_O_2_, mmHg	67 (63–72)
HCO3, mmol/L	29.03 (28.10–30.20)

Data are presented as median (IQR)

*BMI* body mass index, *LTO* long-term oxygen therapy, *GOLD* Global Initiative for Chronic Obstructive Lung Disease, *APACHE* Acute Physiologic and Chronic Health Evaluation, *ABG* arterial blood gases, *NIV* noninvasive ventilation, *PSV* pressure support ventilation, *PEEP *positive end-expiratory pressure, *F*_*i*_*O*_*2*_ inspired oxygen fraction, *HFNC* high-flow nasal cannula

### Ultrasonography measurements

At the time of NIV discontinuation, the median applied PEEP was 7 (6–8) cmH_2_O, while the PSV was 10 (8–12) cmH_2_O, and F_i_O_2_ was 35 (30–42) %. DD was no different among trials (*p* = 0.753). Diaphragm TF (%) was significantly lower with HFNC-30 L/min compared to HFNC-50 and 60 L/min (*p* < 0.001 for all comparisons). Diaphragm TF (%) was not different between HFNC-30 and 40 L/min. All data are displayed in Table 2.

**Table 2:** Ultrasonography measurements and other physiological variables during noninvasive ventilation and high-flow nasal cannula at different flow rates

Variables	NIV	HFNC-30 L/m	HFNC-40 L/m	HFNC-50 L/m	HFNC-60 L/m	*p*-value
DD, cm	20 (17–23)	21 (18–23)	20 (18–24)	20 (18–22)	21 (19–22)	0.753
DTF, %	35.5 (27–35)	35.5 (31–36)	40.0 (35–41)	54.0 (42–59)*,γ	59.0 (48–61)*,γ	< 0.001
RR, breaths/min	21 (19–23)	21 (19–23)	22 (20–23)	21 (20–23)	21 (20–23)	0.611
Comfort (VAS), points	4 (1–5)	8 (8–9)γ	7 (6–7)γ	6 (5–6)*,γ	6 (5–6)*,γ	< 0.001

Data are presented as median (IQR)

*NIV* noninvasive ventilation, *HFNC* high-flow nasal cannula, *DD* diaphragm displacement, *DTF-exp* end-expiration diaphragm thickness, *DTF-insp* end-inspiration diaphragm thickness, *DTF* diaphragm thickening fraction, *RR* respiratory rate, *VAS* visual analog scale

*Indicates *p*< 0.05 compared with HNFC-30. γIndicates *p*< 0.05 compared with NIV

### Other physiological variables

**Fig. 2:**
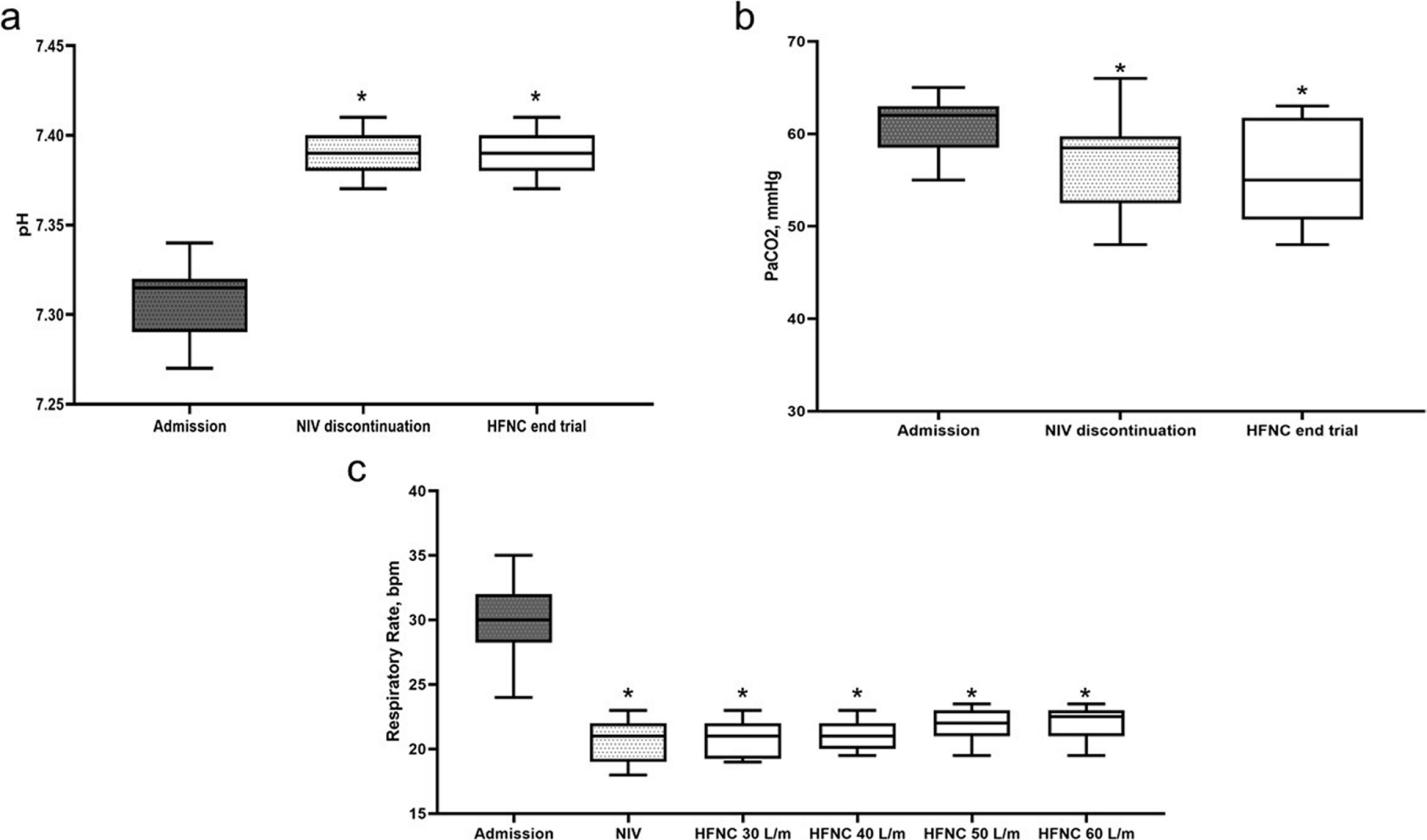
Other physiological variables. **a** pH arterial. **b** P_a_CO_2_ during noninvasive ventilation (NIV) discontinuation and high-flow nasal cannula (HFNC) end trial. **c** Respiratory rate at admission during NIV and HFNC at different flow rates. *Indicates *p* < 0.05 compared with admission

No significant differences were found in arterial pH and P_a_CO_2_ at discontinuation of NIV and at the end of HFNC trials (*p* > 0.050). During HFNC trials, RR remained unchanged without statistically significant differences (*p* = 0.611). The values of the changes in ABG during the interruption of NIV and at the end of the trial with HFNC, along with the trend of RR during the study, are presented in Fig. 2. Interestingly, HFNC at 30 and 40 L/min showed greater comfort during trials.

### Reproducibility of US

ICC were all above 0.96. Coefficients of repeatability ranged around 5–7% for intra-or inter-analyzer repeatability and around 10–13% for intra or inter-observer repeatability.

### Adverse events

The protocol was safely concluded in all 20 patients without any adverse effects. NIV reinstitution within the following 48 h occurred in seven patients (35%).

## Discussion

In this preliminary, physiological prospective pilot study, we evaluated the effects of HFNC at different flow rates on diaphragmatic function in subjects recovering from an AECOPD who had initially been managed and stabilized with NIV. We found that (1) DD and RR were similar across different flow rates during HFNC therapy; (2) diaphragm TF (%) was higher during HFNC-50 and 60 L/min; (3) HFNC improved patient comfort compared to NIV, with the greatest improvement observed at HFNC-30 and 40 L/min; and (4) HFNC flow rate did not provide significant change in arterial pH and P_a_CO_2_.

DD has been extensively studied as an index of diaphragmatic contractile activity [17]. Recent studies indicate that this index does not correlate with other indices of inspiratory effort [28]. In subjects under invasive mechanical ventilation (IMV), DD during an assisted breath represents the combined effect of two forces acting in the same direction: the force generated by the diaphragm’s own contraction and the passive displacement caused by the pressure provided by the ventilator. Although the subjects in our study were not on IMV, they were receiving respiratory support (NIV and HFNC). Longhini et al., in a physiological crossover study involving subjects with acute hypercapnic respiratory failure requiring NIV for 24 h, investigated the impact of transitioning to COT and HFNC-50 L/min. They evaluated diaphragmatic function and other variables [16]. Similar to our findings, they observed no significant difference in DD and RR between the different trials. In contrast to subjects with stable COPD, diaphragm US proved to be a feasible and reliable clinical approach for assessing diaphragm dysfunction in dyspneic hypercapnic acute respiratory failure patients undergoing NIV. Evaluating DD 2 h after NIV initiation was a better predictor of NIV failure compared to pH, P_a_CO_2_, and left expiratory diaphragmatic thickness [20]. In critically ill patients, the most commonly used criterion to indicate diaphragmatic dysfunction is a *DD* < 1–1.1 cm [18, 21]. Recent investigations have reported the presence of diaphragmatic dysfunction in 25–30% of subjects with AECOPD admitted with acute hypercapnic respiratory failure [26, 29]. However, in our study, we did not observe diaphragmatic dysfunction.

Ultrasonography can be used to directly image the diaphragm. Its identification relies on the bright echoes reflected from the attached parietal pleura and peritoneal membranes. Additionally, ultrasonography has been employed to assess the length and thickness of the zone of apposition against the rib cage at different lung volumes. Vivier et al. showed that diaphragm thickening accurately predicts changes in inspiratory muscle effort in response to changes in PSV levels during NIV in subjects after extubation [25]. The DTF (%) could serve as a valuable tool for evaluating diaphragmatic function and its impact on respiratory workload across various scenarios, including during noninvasive respiratory support (NRS) [30]. Additionally, DTF (%) correlates with muscle strength and shortening. In the absence of diaphragm dysfunction, DTF (%) can estimate changes in WOB during NRS, similar findings demonstrated by Umbrello et al. in patients under IMV [28, 30].

The directional changes in DTF (%) following flow increases during HFNC treatment can be explained by findings reported in non-intubated subjects with acute hypoxemic respiratory failure. Higher flow rates linearly improve respiratory drive, end-expiratory lung volume (EELV), lung mechanics, and oxygenation. Meanwhile, effort and minute ventilation decrease exponentially, with most of the beneficial effects achieved at a flow rate of 30 L/min [31]. However, in subjects with COPD, these results may be opposite. Rittayamai et al. investigated the impact of HFNC at flow rates of 10–50 L/ min in COPD subjects who had been previously stabilized with NIV. They encouraged the subjects to keep their mouths closed during HFNC therapy and observed an increase in work of breathing (WOB) at flow rates above 40 L/ min [7]. Similar to our results, with flow rates greater than 50 and 60 L/min, the DTF (%) increased. We hypothesize that patient discomfort, worsening dynamic hyperinflation, or increased resistance to breathing could explain the observed increase in WOB with HFNC at a flow rate of 50 L/min. A study comparing HFNC at 30 L/min vs COT in patients with stable COPD found significant increases in EELV with HFNC. The increase in EELV with HFNC may have aggravated dynamic hyperinflation and effort to breathe in some COPD patients [32]. While comfort during HFNC was higher than NIV, during HFNC-50 and 60 L/min, subjects reported slightly more discomfort in our study. One explanation for this may be due to turbulent airflow, which can increase inspiratory resistance during HFNC therapy with the mouth closed. This turbulent airflow could be generated during the inspiratory phase under HFNC with the mouth closed, particularly when the HFNC flow rate exceeds the inspiratory flow, reverting its direction. This phenomenon could be explained by the findings of Vieira et al. which showed during HFNC at 40 L/min, and inspiratory and expiratory airway resistance was higher with the mouth closed than with the mouth open [33]. The positive airway pressure generated by HFNC with mouth closed can play an important role in terms of clinical effects, and the increased expiratory resistance during HFNC may also contribute to physiological benefits. The increased expiratory resistance during HFNC, especially with the mouth closed, is a mechanism that induces a longer expiratory phase, thereby lowering the RR and minute ventilation [34, 35]. COPD subjects often adopt pursed-lip breathing which helps alleviate expiratory flow limitation and dynamic hyperinflation [36]. In fact, the effect of HFNC on expiratory resistance mimic the pursed-lip breathing effect. In obstructive patients, by contrast, high-flow rates should be used with caution to avoid an excessive increase in airway resistances [7].

Regarding gas exchange, we did not find a significant reduction in arterial pH and P_a_CO_2_ with HFNC. The reduction of anatomical dead space and the consequent elimination of CO_2_ are the mechanism that has been proposed to explain the decrease in P_a_CO_2_ [37]. Indeed, P_a_CO_2_ directly controls the activity of inspiratory muscles alone, and therefore, its reduction may lead to a decrease in diaphragmatic effort. A study by Bräunlich et al. [38] compared HFNC, nasal CPAP, and nasal NIV in 67 hospitalized COPD patients. They found that increasing the flow rate from 20 to 30 L/min improved CO_2_ elimination and reduced P_a_CO_2_. However, considering the significant reduction in RR with similar P_a_CO_2_ levels during NIV and HFNC, it is reasonable to assume that tidal volume is higher under these conditions, unlike COT, as already demonstrated by other authors [16].

In COPD subjects with hypercapnic acute respiratory failure, the rate of NIV discontinuation failure may be relatively high [39]. This inability to maintain unassisted spontaneous breathing depends on an excessive load, which increases diaphragm contraction to an extent that cannot be sustained over time. Longhini et al. reported that although COT caused a marked increase in DTF (%) and HFNC allowed it to remain unchanged. Therefore, according to these results and ours, in subjects recovering from an AECOPD, HFNC could be used during NIV breaks [16, 40].

This study presents limitations:

1. Since it is a sequential study and not a randomized crossover design, the effect of treatment duration on many physiological variables cannot be ruled out.
2. Operator influence on diaphragm ultrasonography was considered. To mitigate this, ultrasound assessments were independently performed by two operators. Consistent with prior research, a high ICC was observed, minimizing the risk of bias.
3. Limitations related to the device prevented the measurement of tidal volume during HFNC and PEEPi levels.
4. The level of PSV and PEEP during NIV in our study was lower compared to levels reported in previous studies, and most patients were stabilized before study inclusion. This factor may introduce a bias favoring HFNC in terms of decreased WOB.

## Conclusion

In subjects recovering from an AECOPD and receiving HFNC, flows above 40 L/min may not offer additional benefits in terms of comfort and decreased respiratory effort. HFNC could be a suitable alternative to COT during breaks off NIV.

## Data Availability

The datasets used and/or analyzed during this study are available from the corresponding author on reasonable request.

## Abbreviations

NIV: Noninvasive ventilation
AECOPD: Acute exacerbation of obstructive pulmonary disease
ETI: Endotracheal intubation
HFNC: High-flow nasal cannula
WOB: Work of breathing
PEEPi: Intrinsic positive end-expiratory pressure
P_a_CO_2_: Arterial carbon dioxide tension
F_i_O_2_: Inspired oxygen fraction
COT: Conventional oxygen therapy
PEEP: Positive end-expiratory pressure
PSV: Pressure support ventilation
DD: Diaphragmatic displacement
DTF: Diaphragm thickening fraction
US: Ultrasonography
RICU: Respiratory intermediate care unit
GOLD: Global initiative for chronic obstructive lung diseases
RR: Respiratory rate
VAS: Visual analog scale
APACHE II: Acute Physiology and Chronic Health Evaluation II
ICC: Intra-class correlation coefficient
IMV: Invasive mechanical ventilation
EELV: End-expiratory lung volume
